# Diseases of the Antrum

**Published:** 1873-12

**Authors:** A. Vans Best


### SELECTED ARTICLES.

---

### ARTICLE VI.

### *Diseases of the Antrum.*

BY A. VANS BEST, M. D., F. R. C. S.

The diseases of the cavity of the antrum proper must be kept, in the surgical description and treatment, separate from those which have their rise in the alveolar process—such as epulis or ostitis—terminating in necrosis, &c. The diagnosis of disease in the interior of the antrum is, at first, very difficult, and the symptoms about to be described fail to point out, with certainty, with what disease the surgeon has to contend.

In the first place, pain, a feeling of weight, and "neuralgia" are complained of, and a slight puffiness of the cheek is observed. The nostril on the side effected is generally dry, and on examining the mouth there will usually be found disease of the bicuspid teeth, with, probably, necrosis of the root of one of them, which may or may not have found its way into the antrum. Under these circumstances I have never seen any enlargement of the antrum, although the cheek is usually puffed. The proper treatment, of course, is to extract the dead portion of the tooth, make a free opening with the trocar, and wash out the cavity with some weak disinfecting solution. Should it be impossible to ascertain, in a mouth filled with stumps, which is most to blame, I prefer passing a full-sized flat trocar above the root of the second bicuspid.

A second class of cases occurs in which we have dropsy, or pseudo-dropsy of the antrum—in my experience always associated with non-malignant polypus, exactly similar to the fibroid polypus of the nose. In these cases little pain is felt, and the only uneasiness complained of is a feeling of stuffiness in the head, weight in the affected part, and lachrymation from the eye of the side affected. Should the opening from the antrum into the nose be closed, the pressure from the accumulated fluid soon thins the anterior wall, and the cheek, on being pressed upon, gives a most characteristic crackling sensation. The face being swollen, it is generally then that people of the middle and lower classes first apply for advice. In cases such as these there is no necessity for the removal of the antrum; the proper treatment I consider to be to divide, if necessary, the upper lip, tying the coronary arteries, pulling up the flap, and with a strong scalpel, dividing and removing the whole of the attenuated bone. Probably a considerable amount of serum will escape, and most likely a polypus will be found in the cavity. The only risk attending this operation is hæmorrhage from a wide-based polypus, and this is easily controlled by the actual cautery, or plugging with a strip of lint dipped in perchloride of iron with glycerine.

Osteo-sarcoma and enchondroma of the upper jaw require complete ablation. There is no difficulty in the diagnosis, as the tumor is firmer than shcirrhus, rough and irregular in outline, and slow of growth. On opening the front part of the antrum, should the tumor be found to present the character of encephaloma, it would be worse than useless to attempt to extract it, as it would certainly recur from the numerous processes extending toward the back of the orbit and base of the skull.

In schirrhus, however, it appears to be different. The diagnostic points of schirrus of the antrum I think may be described as follows:—Hereditary predisposition; advanced age; constant dull pain in the part, with occasional stabbing pain; the speedy appearance of a swelling in the cheek and palate, which may in time be also felt with the finger at the posterior nares; and, as the disease advances, the eye may be extruded. Excessive pain generally prevents the sufferer from sleeping, and this is the principal reason that would lead the surgeon to remove the superior maxilla—not so much to cure the disease as to give comfort to the patient and to prolong a life which would certainly speedily come to a close from the wearing suffering the disease entails. The operation, however, of the removal of the whole antrum is generally held to be unjustifiable should any enlargement of the submaxillary glands exist, still more so should any head symptoms show that processes of the tumor extend towards the base of the brain.

Cases of abscess of the antrum treated by perforation, and completed by cure, are far from uncommon. Fibrous tumor of the antrum I have twice removed by cutting through the attenuated bone with a strong scalpel, and stopping the hæmorrhage with a long strip of lint firmly plugged in the cavity; in neither of those cases did I divide the lip.

Tho entire removal of the right superior maxilla, together with the pterygoid process and palate bone, is a much more serious affair, both for the patient and the surgeon, and I am glad to place on record an instance of its successful performance.

Mrs. R——, aged sixty-three, sanguine and healthy-looking, had from the month of May, 1872, pain in the right antrum, which gradually increased in severity during three months. She thought it was rheumatic. No swelling appeared till the end of June, when she became sleepless night, and a dull aching pain lasted all day. She had lost all her teeth on that side, with one exception, and that was removed without benefit. I first saw this case about the beginning of July, when there was fullness of cheek, and a lobulated expansion of the hard palate, of the right side; and a tolerably hard tumor could be felt at the back of the posterior nares; great persistent pain was complained of; there were no enlarged glands. An exploratory puncture was made by her usual medical attendant, with a negative result. I subsequently say her, arranged matters, and at her urgent desire, operated on Saturday, September 21st.

The patient being supported in a chair, with a sheet tied round her, chloroform was gradually administered. I divided the upper lip into the right nostril; tied the coronary arteries; entered my scalpal over the right malar bone, cutting to the bone, across to the nasal process of the superior maxilla, then down the side of the nose, round the ala, to join the first incision. I quickly reflected the whole flap, holding it firmly to compress the vessels, and dissecting free of a nodule of tumor prominent in the anterior wall. I then tied what vessels bled; cut with a cutting pliers the malar bone above and below, completing the section, as also the external orbital angle and the nasal, and the internal angle of the orbit, with a Liston's forceps. With a pruning-claw nippers, the hard palate was easily divided. After separating the eye from the orbital plate, I found the lion forceps would break up the tumor; so I used a long flat-bladed lithotomy forceps, placing one blade in the orbit, and dividing the pterygoids and other attachments, succeeded in wrenching the whole mass away. Immediately on its removal, a very large jet of blood sprang from the deepest corner of the wound; this was immediately controlled by the finger, and arrested by two applications of the actual cautery. This probably was the internal maxillary greatly enlarged; but its situation was so deep at the tip of the petrous portion of the temporal and body of the sphenoid bone, that it is impossible to be sure of the vessel. A few parts of the soft palate and cheek required a little trimming, and several small vessels were ligatured ; long strips of lint damped with tincture of muriate of iron were pressed to the very base of the skull, and the wound united with gold wire sutures, except the free mucous membrane of the lip, which was stitched.

The patient soon rallied, and slept almost continuously for two days, being only wakened to have her mouth syringed and to get milk, beef tea, and ice. On the third day all the strips of lint were removed, without any hæmorrhage; the greatest attention was paid to syringing the whole wound with diluted Condy's fluid, and at times, carbolic acid lotion. Five weeks after the operation all the external wound was healed, with the exception of two small holes in the transverse incision ; and the patient returned home quite relieved of all her former suffering, and with a fair chance of life being prolonged for some years.

In doing this operation again, I would not cut quite so close to the orbit, as the tissues are not very vital there. There was no sloughing, but, notwithstanding a free allowance of port wine, bark and whisky, these portions would not unite by the first intention.

The tumor presents the following appearance :—It is of the size, and somewhat of the shape of a large lemon. Below half the palate ; posteriorly, the mucous membrane of the posterior nares and pharynx ; internally, the turbinated bones ; externally and laterally, a long tongue that has extended into the spheno maxillary fossa; anteriorly, a rounded protrusion through the wall ; superiorly, the orbital plate. It is quite solid after immersion in spirits ; and microscopically, is an admirable example of fibrous schirrhus.

The patient can speak wonderfully well, sleeps, eats soft food, and is in every way comfortable. She left for her home within six weeks of the operation.

I was most ably assisted by Dr. A. Forbes ; Dr. Scott, of H. M's Navy ; Mr. Moir, the house surgeon of the Royal Infirmary, Aberdeen ; and Dr. Inglis, who most judiciously kept the patient sufficiently under chloroform. There was no trouble from blood getting into the larynx.—*London Lancet.*

